# 4-(4-Nitro­benz­yl)pyridine

**DOI:** 10.1107/S1600536813017145

**Published:** 2013-06-26

**Authors:** Deeb Taher, Firas F. Awwadi, Mohammed H. Kailani

**Affiliations:** a Department of Chemistry, The University of Jordan, Amman 11942, Jordan

## Abstract

The title compound, C_12_H_10_N_2_O_2_, has a twisted conformation, with a dihedral angle between the planes of the pyridine and benzene rings of 78.4 (2)°. The nitro group is coplanar with the attached benzene ring within experimental error. The mol­ecules form centrosymmetric dimers *via* C_ar_—H⋯O inter­actions (H⋯O = 2.49 Å) and the dimers are π-stacked along the *b* axis [the separation between ring centroids is 3.788 (2) Å].

## Related literature

For adducts of the title compound with different organic acids, see: Smith *et al.* (1997 ▶); Smith & Wermuth (2010 ▶, 2013 ▶). For a zinc complex of the title compound, see: Smith *et al.* (2011 ▶). For the analysis of π-stacking inter­actions, see: Dolomanov *et al.* (2009 ▶).
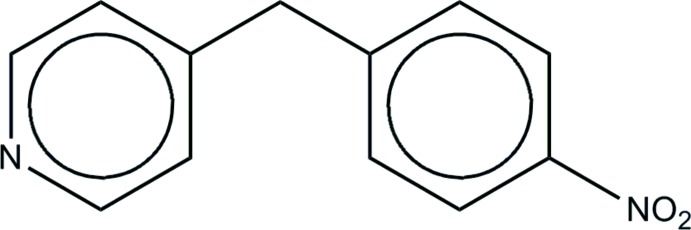


## Experimental

### 

#### Crystal data

C_12_H_10_N_2_O_2_
*M* *_r_* = 214.22
Monoclinic, 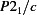

*a* = 11.4138 (9) Å
*b* = 6.1241 (5) Å
*c* = 15.5812 (13) Å
β = 104.561 (9)°
*V* = 1054.13 (15) Å^3^
*Z* = 4
Mo *K*α radiation
μ = 0.09 mm^−1^
*T* = 293 K
0.4 × 0.2 × 0.15 mm

#### Data collection

Agilent Xcalibur Eos diffractometer
Absorption correction: multi-scan (*CrysAlis PRO*; Agilent, 2011 ▶) *T*_min_ = 0.770, *T*_max_ = 1.000
4351 measured reflections
2136 independent reflections
1514 reflections with *I* > 2σ(*I*)
*R*_int_ = 0.018

#### Refinement

*R*[*F* ^2^ > 2σ(*F* ^2^)] = 0.047
*wR*(*F* ^2^) = 0.106
*S* = 1.03
2136 reflections
145 parameters
H-atom parameters constrained
Δρ_max_ = 0.12 e Å^−3^
Δρ_min_ = −0.15 e Å^−3^

### 

Data collection: *CrysAlis PRO* (Agilent, 2011 ▶); cell refinement: *CrysAlis PRO*; data reduction: *CrysAlis PRO*; program(s) used to solve structure: *SHELXS97* (Sheldrick, 2008 ▶); program(s) used to refine structure: *SHELXL97* (Sheldrick, 2008 ▶); molecular graphics: *XP* in *SHELXTL* (Sheldrick, 2008 ▶); software used to prepare material for publication: *SHELXTL* (Sheldrick, 2008 ▶).

## Supplementary Material

Crystal structure: contains datablock(s) I, global. DOI: 10.1107/S1600536813017145/ld2106sup1.cif

Structure factors: contains datablock(s) I. DOI: 10.1107/S1600536813017145/ld2106Isup2.hkl

Supplementary material file. DOI: 10.1107/S1600536813017145/ld2106Isup3.cml

Additional supplementary materials:  crystallographic information; 3D view; checkCIF report

## Figures and Tables

**Table 1:** Hydrogen-bond geometry (Å, °)

*D*—H⋯*A*	*D*—H	H⋯*A*	*D*⋯*A*	*D*—H⋯*A*
C9—H9*A*⋯O2^i^	0.93	2.49	3.302 (2)	146

Symmetry code: (i) 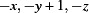.

**Fig. 1.**
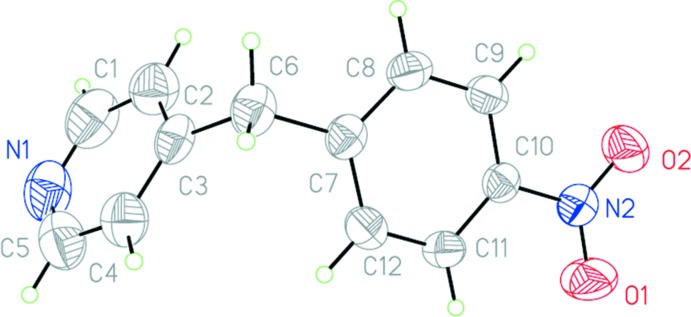
Molecular unit of the title compound. Thermal ellipsoids are shown at 50% probability.

**Fig. 2.**
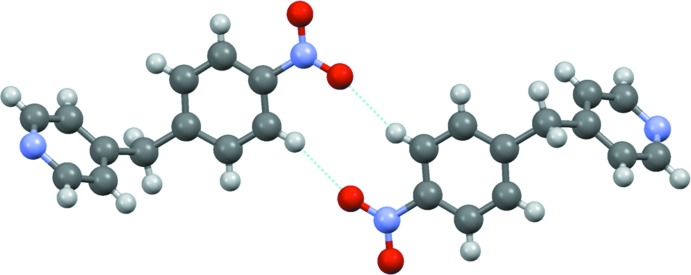
Structure of the cyclic dimer.

**Fig. 3.**
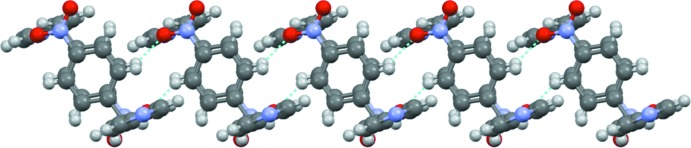
Illustration of the ribbon structure of the title compound.

**Fig. 4.**
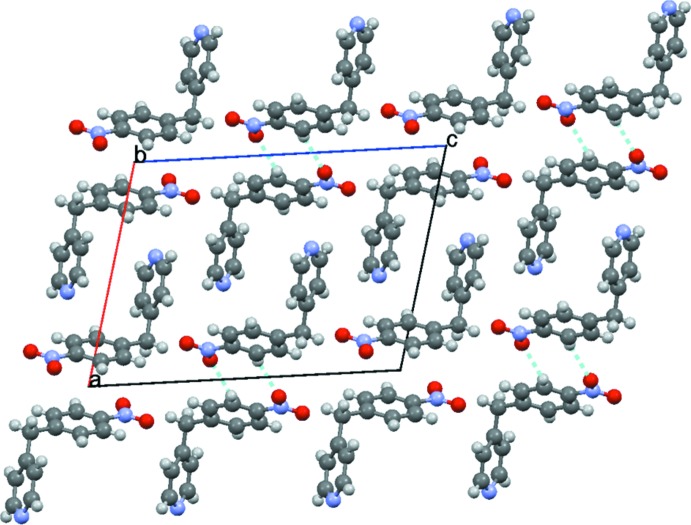
Illustration of the three dimensional structure of the title compound viewed along the b-axis.

## supplementary crystallographic information

### Comment

X-Ray structure of the title compound was never reported before in its non-coordinated form, even though several works have been published on it's pyridiunium salts/adducts. The adducts with carboxilic acids were reported for 4-aminobenzoic (Smith *et al.*, 1997) and 5-nitrosalicylic acid (Smith & Wermuth, 2010). Recently, a structure of an adduct with 3-carboxy-4-hydroxybenzenesulfonic acid was also determined (Smith & Wermuth, 2013). The structures of the adducts are dominated by *N*(pyridine)—H···O hydrogen bonding interactions. In addition, X-ray structure of a zinc complex of the title compound (Diiodidobis[4-(4-nitrobenzyl)pyridine-_κ_N^1^]zinc) has also been determined (Smith *et al.*, 2011).

The title compound (Fig. 1) gives colorless crystals. The angle between the planes of benzene and pyridine rings is 78.43° and the nitro group is coplanar with the benzene ring. The two aromatic planes are twisted relative to each other, which result in reduction of molecular symmetry from C_s_ to C_1_: the dihedral angle C2—C3···C7—C8 is 30.5 (2)°. Two molecular units of the title compound inter-associate through duplex C9—H···O2 hydrogen bonds to form a cyclic dimer (Fig. 2 and Table 1). Then, these dimers are stacked *via*π···π interactions between benzene rings to form ribbon structure extending parallel to *b-*axis (Fig. 3); the angle between the two planes, centroid-centroid distance and shift distance are 0°, 3.788 Å and 1.613 Å, respectively, as determined by Olex2 program package (Dolomanov *et al.*, 2009). Subsequently, these ribbons are interdigitated to form the final three-dimensional structure (Fig. 4).

The nitro group of the title compound, was found to be a major factor in determining the interactions in the crystal form, unlike in the previously published structures where the pyridinic nitrogen was the main driving force for amolecular association.

### Experimental

Crystals of the title compound were obtained by dissolving 1 mmol of 4-(4-nitrobenzyl) pyridine in 30 ml of hot 96% ethanol. Partial evaporation of the hot-filtered solution at room temperature yielded colourless crystals from which a block section was cleaved for the X-ray analysis.

### Refinement

The structure was solved by direct methods and refined by least squares method on F2 using the *SHELXTL* program package. All atoms were refined anisotropically. Hydrogen atoms were placed at the calculated positions using a riding model with C(aromatic)— H = 0.95 Å and *U*_iso_(H) = 1.2Ueq(C), and with C(aliphatic)—H = 0.98 Å and *U*_iso_(H) = 1.5Ueq(C). refinement details)

### Crystal data

**Table:**

C_12_H_10_N_2_O_2_	*F*(000) = 448
*M**_r_* = 214.22	*D*_x_ = 1.350 Mg m^−^^3^
Monoclinic, *P*2_1_/*c*	Mo *K*α radiation, λ = 0.71073 Å
Hall symbol: -P 2ybc	Cell parameters from 949 reflections
*a* = 11.4138 (9) Å	θ = 3.3–29.0°
*b* = 6.1241 (5) Å	µ = 0.09 mm^−^^1^
*c* = 15.5812 (13) Å	*T* = 293 K
β = 104.561 (9)°	Needle, white
*V* = 1054.13 (15) Å^3^	0.4 × 0.2 × 0.15 mm
*Z* = 4	

### Data collection

**Table:**

Agilent Xcalibur Eos diffractometer	2136 independent reflections
Radiation source: Enhance (Mo) X-ray Source	1514 reflections with *I* > 2σ(*I*)
Graphite monochromator	*R*_int_ = 0.018
Detector resolution: 16.0534 pixels mm^-1^	θ_max_ = 26.3°, θ_min_ = 3.6°
ω scans	*h* = −13→14
Absorption correction: multi-scan (*CrysAlis PRO*; Agilent, 2011)	*k* = −7→6
*T*_min_ = 0.770, *T*_max_ = 1.000	*l* = −19→19
4351 measured reflections	

### Refinement

**Table:**

Refinement on *F*^2^	Primary atom site location: structure-invariant direct methods
Least-squares matrix: full	Secondary atom site location: difference Fourier map
*R*[*F*^2^ > 2σ(*F*^2^)] = 0.047	Hydrogen site location: inferred from neighbouring sites
*wR*(*F*^2^) = 0.106	H-atom parameters constrained
*S* = 1.03	*w* = 1/[σ^2^(*F*_o_^2^) + (0.0401*P*)^2^ + 0.1216*P*] where *P* = (*F*_o_^2^ + 2*F*_c_^2^)/3
2136 reflections	(Δ/σ)_max_ < 0.001
145 parameters	Δρ_max_ = 0.12 e Å^−^^3^
0 restraints	Δρ_min_ = −0.15 e Å^−^^3^

### Special details

**Table:**

Experimental. Absorption correction CrysAlis PRO (Agilent, 2011). Empirical absorption correction using spherical harmonics, implemented in SCALE3 ABSPACK scaling algorithm.
Geometry. All e.s.d.'s (except the e.s.d. in the dihedral angle between two l.s. planes) are estimated using the full covariance matrix. The cell e.s.d.'s are taken into account individually in the estimation of e.s.d.'s in distances, angles and torsion angles; correlations between e.s.d.'s in cell parameters are only used when they are defined by crystal symmetry. An approximate (isotropic) treatment of cell e.s.d.'s is used for estimating e.s.d.'s involving l.s. planes.
Refinement. Refinement of *F*^2^ against ALL reflections. The weighted *R*-factor *wR* and goodness of fit *S* are based on *F*^2^, conventional *R*-factors *R* are based on *F*, with *F* set to zero for negative *F*^2^. The threshold expression of *F*^2^ > σ(*F*^2^) is used only for calculating *R*-factors(gt) *etc*. and is not relevant to the choice of reflections for refinement. *R*-factors based on *F*^2^ are statistically about twice as large as those based on *F*, and *R*- factors based on ALL data will be even larger.

### Fractional atomic coordinates and isotropic or equivalent isotropic displacement parameters (Å^2^)

**Table:**

	*x*	*y*	*z*	*U*_iso_*/*U*_eq_	
N2	0.13729 (12)	0.2522 (2)	0.13717 (9)	0.0459 (4)	
C10	0.15049 (13)	0.1215 (2)	0.06150 (10)	0.0361 (4)	
C9	0.11079 (14)	0.2054 (3)	−0.02263 (11)	0.0413 (4)	
H9A	0.0757	0.3433	−0.0317	0.050*	
C7	0.17562 (14)	−0.1248 (3)	−0.08069 (10)	0.0391 (4)	
O2	0.09206 (12)	0.4333 (2)	0.12370 (9)	0.0633 (4)	
C11	0.20080 (14)	−0.0837 (3)	0.07671 (11)	0.0410 (4)	
H11A	0.2258	−0.1390	0.1340	0.049*	
C8	0.12404 (14)	0.0808 (3)	−0.09329 (11)	0.0427 (4)	
H8A	0.0978	0.1361	−0.1505	0.051*	
C12	0.21313 (14)	−0.2047 (3)	0.00541 (11)	0.0426 (4)	
H12A	0.2473	−0.3433	0.0149	0.051*	
C6	0.19074 (15)	−0.2601 (3)	−0.15842 (11)	0.0496 (5)	
H6A	0.1457	−0.3953	−0.1607	0.059*	
H6B	0.1576	−0.1809	−0.2130	0.059*	
O1	0.17074 (16)	0.1747 (2)	0.21073 (9)	0.0835 (5)	
C3	0.32183 (15)	−0.3119 (3)	−0.15136 (10)	0.0445 (4)	
C4	0.37349 (17)	−0.5080 (3)	−0.11888 (12)	0.0555 (5)	
H4A	0.3263	−0.6168	−0.1028	0.067*	
N1	0.56897 (15)	−0.3995 (3)	−0.13144 (12)	0.0712 (5)	
C2	0.39748 (17)	−0.1617 (3)	−0.17558 (13)	0.0610 (5)	
H2A	0.3676	−0.0274	−0.1992	0.073*	
C5	0.49444 (19)	−0.5428 (4)	−0.11034 (14)	0.0670 (6)	
H5A	0.5263	−0.6770	−0.0881	0.080*	
C1	0.51831 (19)	−0.2128 (4)	−0.16440 (15)	0.0731 (6)	
H1B	0.5676	−0.1088	−0.1812	0.088*	

### Atomic displacement parameters (Å^2^)

**Table:**

	*U*^11^	*U*^22^	*U*^33^	*U*^12^	*U*^13^	*U*^23^
N2	0.0485 (9)	0.0470 (9)	0.0436 (9)	0.0015 (7)	0.0142 (7)	−0.0001 (7)
C10	0.0327 (8)	0.0392 (9)	0.0376 (9)	−0.0024 (7)	0.0108 (7)	0.0001 (7)
C9	0.0387 (9)	0.0401 (9)	0.0448 (10)	0.0031 (7)	0.0098 (8)	0.0074 (8)
C7	0.0308 (8)	0.0477 (10)	0.0398 (9)	−0.0043 (7)	0.0104 (7)	−0.0016 (8)
O2	0.0808 (10)	0.0477 (8)	0.0618 (9)	0.0179 (7)	0.0189 (7)	−0.0018 (7)
C11	0.0430 (9)	0.0428 (9)	0.0363 (9)	0.0032 (8)	0.0086 (8)	0.0070 (8)
C8	0.0424 (9)	0.0498 (10)	0.0350 (9)	−0.0003 (8)	0.0082 (8)	0.0064 (8)
C12	0.0396 (9)	0.0385 (9)	0.0494 (10)	0.0037 (7)	0.0109 (8)	0.0026 (8)
C6	0.0413 (10)	0.0621 (11)	0.0455 (10)	−0.0024 (8)	0.0113 (8)	−0.0088 (9)
O1	0.1380 (14)	0.0749 (10)	0.0378 (8)	0.0326 (10)	0.0224 (8)	0.0078 (7)
C3	0.0436 (9)	0.0551 (11)	0.0361 (9)	−0.0042 (9)	0.0126 (8)	−0.0120 (8)
C4	0.0519 (11)	0.0561 (11)	0.0601 (12)	−0.0023 (9)	0.0172 (10)	−0.0061 (10)
N1	0.0481 (10)	0.0950 (14)	0.0719 (12)	0.0042 (10)	0.0176 (9)	−0.0145 (11)
C2	0.0540 (12)	0.0668 (13)	0.0653 (13)	−0.0035 (10)	0.0211 (10)	0.0019 (11)
C5	0.0585 (13)	0.0708 (14)	0.0692 (14)	0.0117 (12)	0.0114 (11)	−0.0119 (11)
C1	0.0535 (13)	0.0956 (17)	0.0759 (15)	−0.0169 (13)	0.0271 (12)	−0.0053 (14)

### Geometric parameters (Å, º)

**Table:**

N2—O1	1.2101 (17)	C6—C3	1.506 (2)
N2—O2	1.2194 (17)	C6—H6A	0.9700
N2—C10	1.464 (2)	C6—H6B	0.9700
C10—C9	1.374 (2)	C3—C4	1.377 (2)
C10—C11	1.377 (2)	C3—C2	1.378 (2)
C9—C8	1.379 (2)	C4—C5	1.370 (3)
C9—H9A	0.9300	C4—H4A	0.9300
C7—C8	1.383 (2)	N1—C5	1.320 (3)
C7—C12	1.391 (2)	N1—C1	1.325 (3)
C7—C6	1.513 (2)	C2—C1	1.382 (3)
C11—C12	1.372 (2)	C2—H2A	0.9300
C11—H11A	0.9300	C5—H5A	0.9300
C8—H8A	0.9300	C1—H1B	0.9300
C12—H12A	0.9300		
			
O1—N2—O2	122.59 (15)	C3—C6—H6A	109.3
O1—N2—C10	118.47 (14)	C7—C6—H6A	109.3
O2—N2—C10	118.93 (14)	C3—C6—H6B	109.3
C9—C10—C11	121.90 (15)	C7—C6—H6B	109.3
C9—C10—N2	119.20 (14)	H6A—C6—H6B	108.0
C11—C10—N2	118.89 (14)	C4—C3—C2	116.31 (17)
C10—C9—C8	118.60 (15)	C4—C3—C6	122.41 (16)
C10—C9—H9A	120.7	C2—C3—C6	121.27 (17)
C8—C9—H9A	120.7	C5—C4—C3	119.94 (18)
C8—C7—C12	118.32 (15)	C5—C4—H4A	120.0
C8—C7—C6	121.02 (15)	C3—C4—H4A	120.0
C12—C7—C6	120.66 (15)	C5—N1—C1	115.11 (18)
C12—C11—C10	118.48 (15)	C3—C2—C1	119.2 (2)
C12—C11—H11A	120.8	C3—C2—H2A	120.4
C10—C11—H11A	120.8	C1—C2—H2A	120.4
C9—C8—C7	121.25 (15)	N1—C5—C4	124.7 (2)
C9—C8—H8A	119.4	N1—C5—H5A	117.6
C7—C8—H8A	119.4	C4—C5—H5A	117.6
C11—C12—C7	121.44 (15)	N1—C1—C2	124.7 (2)
C11—C12—H12A	119.3	N1—C1—H1B	117.7
C7—C12—H12A	119.3	C2—C1—H1B	117.7
C3—C6—C7	111.59 (13)		
			
O1—N2—C10—C9	178.75 (15)	C6—C7—C12—C11	179.59 (14)
O2—N2—C10—C9	−0.5 (2)	C8—C7—C6—C3	119.47 (17)
O1—N2—C10—C11	−0.3 (2)	C12—C7—C6—C3	−60.7 (2)
O2—N2—C10—C11	−179.55 (15)	C7—C6—C3—C4	98.12 (19)
C11—C10—C9—C8	−1.2 (2)	C7—C6—C3—C2	−80.4 (2)
N2—C10—C9—C8	179.84 (13)	C2—C3—C4—C5	1.5 (3)
C9—C10—C11—C12	1.2 (2)	C6—C3—C4—C5	−177.18 (16)
N2—C10—C11—C12	−179.81 (13)	C4—C3—C2—C1	−1.4 (3)
C10—C9—C8—C7	0.2 (2)	C6—C3—C2—C1	177.24 (17)
C12—C7—C8—C9	0.6 (2)	C1—N1—C5—C4	−1.2 (3)
C6—C7—C8—C9	−179.57 (14)	C3—C4—C5—N1	−0.1 (3)
C10—C11—C12—C7	−0.3 (2)	C5—N1—C1—C2	1.3 (3)
C8—C7—C12—C11	−0.6 (2)	C3—C2—C1—N1	0.0 (3)

### Hydrogen-bond geometry (Å, º)

**Table:**

*D*—H···*A*	*D*—H	H···*A*	*D*···*A*	*D*—H···*A*
C9—H9*A*···O2^i^	0.93	2.49	3.302 (2)	146

Symmetry code: (i) −*x*, −*y*+1, −*z*.
